# Determinants of stillbirth among mothers who gave birth at Bishoftu General Hospital, Ethiopia: using a Bayesian logistic regression model

**DOI:** 10.3389/fgwh.2024.1441636

**Published:** 2024-12-06

**Authors:** Yeshi Regassa, Hunde Lemi, Tesfaye Getachew Charkos

**Affiliations:** School of Public Health, Adama Hospital Medical College, Adama, Ethiopia

**Keywords:** stillbirth, pregnancy, determinants, Bayesian, Ethiopia

## Abstract

**Introduction:**

Stillbirth remains a major public health problem in developing countries due to low health coverage and services. Approximately two million stillbirths occur every year; in particular, stillbirths are highly prevalent in low- and middle-income countries such as Ethiopia. However, there is scarce information available in Ethiopia regarding the determinants of stillbirth.

**Methods:**

A facility-based, unmatched case-control study was conducted at Bishoftu General Hospital from April 1 to April 30, 2023. A systematic random sampling technique was used to select samples of the study subjects. The collected data were entered into Epi-info version 7.2. A Bayesian logistic regression model was used in this study, with a 95% Credible interval to determine the association between independent variables and stillbirth. All analyses were performed using STATA version 17 software.

**Results:**

A total of 403 (81 cases and 322 controls) participants were included in the study. The average age of participants was 26 years (SD: ±5.03). According to the adjusted model, mothers who attained a middle school [adjusted odds ratio [AOR] = 0.23; 95% credible interval [CrI]: 0.11, 0.43], diploma and above (AOR = 0.25; 95% CrI: 0.12, 0.46), rural residence (AOR = 2.55; 95% CrI: 1.11, 4.99), married women (AOR = 0.46; 95% CrI: 0.20, 0.93), ANC visits more than 4 (AOR = 0.35; 95% CrI: 0.17, 0.63), history of prior stillbirth (AOR = 8.71; 95% CI: 3.22, 17.69), previous history of abortion (AOR = 3.29; 95% CI: 1.13, 7.666), duration of labor more than 24 h (AOR = 3.71; 95% CI: 1.28, 7.83); normal birth weight (AOR = 0.39; 95% CI: 0.15, 0.57) were significantly associated with stillbirth.

**Conclusions:**

This study revealed that maternal education, rural residence, marital status, history of prior stillbirth, ANC visit, previous history of abortion, duration of labor, and birth weight were identified as determinants of stillbirth. Pregnant mothers should be identified early and given appropriate care, including comprehensive prenatal care and other maternal services.

## Introduction

Stillbirth is indeed a tragic and often underexplored aspect of pregnancy outcomes; it is a common cause of perinatal mortality (1). In 2015, the World Health Organization estimated that 2.4 to 3.0 million stillbirths occur each year globally (2). These figures indicate a 19% decrease in the number of stillbirths since 2000. The vast majority (98%) of stillborn neonates occur in low- and middle-income countries, particularly in sub-Saharan Africa and South Asia (77%) (3). In developing countries, stillbirth remains a major health problem, primarily due to poor-quality care during pregnancy and childbirth (4). Recently, the practice of skilled deliveries has increased in Ethiopia, but the reduction in stillbirths has not been as significant as required (5). Despite achieving marked improvements in many health indicators, including reductions in maternal and child mortality over the last decade, Ethiopia has remained one of the countries with the highest stillbirth rates (1, 6–9).

The prevalence of stillbirths in developing countries remains significantly high (10–12), particularly in Sub-Saharan Africa, including Ethiopia (13, 14). Studies show that Ethiopia is among the countries with the highest stillbirth rates globally, with recent figures indicating around 11 to 12 stillbirths per 1,000 live births (15, 16). A meta-analysis of 21 studies on stillbirth in Ethiopia revealed a pooled prevalence of 7.84% (17). Similarly, a facility-based cross-sectional study reported that the proportion of stillbirths in Ethiopia ranges from 3% to 10% (18–20).

Studies have identified several factors associated with stillbirth, including maternal infections (21–23), asphyxia (22), chronic illnesses (21, 24), and previous preterm births (21, 23, 25, 26). Other risk factors include prolonged labor (21, 25), green or light brown liquor (25), multiple pregnancies (27, 28), maternal age (5, 25, 28), poor antenatal care utilization (9, 21, 26, 29), mode of delivery (9, 25), place of delivery (28), Do not know danger signs during pregnancy (27). low educational levels (23, 26–28), rural residents (21, 27, 28), short inter pregnancy interval (21), gender of child (27), Body mass index (28), poor family (26), and family size (25). Additional contributors include conditions such as anemia (28), uterine rupture (21–23), placental abruption (22, 30), antepartum hemorrhage (26, 30), hypertensive disorders during pregnancy (22, 24), and small for gestational age babies (5, 24, 26, 30, 31). These factors highlight the complex interplay of medical, socio-economic, and environmental influences on stillbirth outcomes.

Ethiopia is implementing the National Newborn and Child Survival Strategy (32) and the Maternal and Child Health Quality Improvement Plan (33) to reduce neonatal mortality and prevent stillbirths. These initiatives prioritize skilled birth attendance, quality antenatal and emergency care, and education on stillbirth risks. Additional efforts include community-based health education, early detection of pregnancy complications, and improved access to obstetric care in rural areas to encourage proactive health-seeking behaviors (32, 34, 35), which have yielded favorable outcomes in reducing stillbirth and neonatal mortality. However, despite these efforts, stillbirth and neonatal mortality rates remain a significant public health issue, underscoring the urgent need for further research to identify healthcare gaps and improve maternal and neonatal health interventions.

Despite various strategies being implemented in Ethiopia, the causes of most stillbirths remain unknown, with studies identifying a poor referral system as a major contributor (36–40). Evidence indicates that many stillbirths are preventable (41–44). Given the high rate of stillbirths at Bishoftu General Hospital, which receives high-risk referrals from under-resourced centers, we conducted a case-control study comparing stillbirths with live births to identify risk factors, care gaps, and socio-economic and medical contributors. The findings will help guide targeted interventions, improve prenatal care, enhance outcomes for high-risk pregnancies, reduce the burden on tertiary hospitals through early community-level care, and inform policy and resource allocation to improve maternal and neonatal health.

## Methods

### Study area and design

A facility-based unmatched case-control study was conducted from April 1 to April 30, 2023, at Bishoftu General Hospital, Ethiopia. The population is composed of multiple ethnic groups and mixed cultures. Bishoftu town has a total population of 234,970, approximately 50% of which are female. Women of reproductive age account for 51,998 of the total population.

### Inclusion and exclusion criteria

The inclusion criteria for patients (cases) were as follows: (1) pregnant women who delivered at Bishoftu General Hospital and whose delivery outcomes were documented; (2) pregnant women who were followed at the Bishoftu General Hospital during pregnancy; and (3) delivery outcome of greater than or equal to twenty-eight weeks of pregnancy. Participants with incomplete data or those transferred from other health centers were excluded from this analysis. The study participants were selected using a systematic random sampling method. The data were extracted by two nurses and degree midwives.

### Sample size determination

The sample size for this study was calculated using the double population proportion formula in Epi-Info version 7.2, based on the occurrence of stillbirth. Baseline determinants, including low birth weight, mode of admission, and duration of labor, were drawn from similar studies conducted at Hawassa University Hospital (45) and Bale Zone hospitals (8). The assumptions for sample size calculation included a two-sided 95% confidence interval (1.96), 80% power, and 5% types I error (α = 0.05). The final calculated sample size was 403, consisting of 81 cases and 322 controls, with a 4:1 case-to-control ratio.

### Study variables and measurements

The dependent variable in this study was stillbirth status. Independent variables included in this study were sociodemographic factors, such as age, marital status, residence, educational status, occupation, maternal medical-related factors (diabetes, hypertension, HIV, TB, congenital factors, maternal rh factors, birthweight, iron, and foliate supplement use), and obstetric factors (history of stillbirth, history of abortion, mode of delivery, labor duration).

### Data analysis

The descriptive statistics of frequency and percentage were used for categorical variables. The mean **(**standard deviation**)** was used for normally distributed continuous variables. We utilized Bayesian logistic regression over the frequentist approach due to several advantages: (1) Bayesian analysis provides more reliable estimates with a small sample size; (2) it allows for the incorporation of prior knowledge, enhancing estimates when relevant data or expert insights are available; (3) unlike the *p*-value-based frequentist approach, Bayesian models provide credible intervals for direct probabilistic interpretation and more robust uncertainty quantification through posterior distributions, *p*-value may lead to imprecise evidence, as it depends on the sample size; and (4) Bayesian logistic regression supports complex model structures, reduces overfitting, and enhances predictive stability, making it ideal for our study, which focuses on interpretability, uncertainty management, and the integration of prior insights. In a Bayesian model, specifying prior information is essential (46, 47). We opted for a non-informative prior to avoid subjective bias and ensure that the analysis was based on our study data. Although relevant studies from Ethiopia exist, variability in the available prior data could lead to overestimating its influence. Therefore, we chose not to use an informative prior, ensuring the analysis remained objective and data-driven. Consequently, we assumed prior information for each variable's coefficient to be normally distributed with a zero mean and a variance of 10,000. We conducted 10,000 iterations for estimation with a burn-in of 5,000, which showed good convergence during the analysis. All analyses were conducted using STATA version 17 software (StataCorp. 2021).

### Operational definition

**Stillbirth:** is defined as a baby born at or after 28 weeks of gestation who does not show any sign of life after delivery, breath, or shows any other signs of life (48, 49).

**History of poor obstetric outcome:** mothers who had a history of LBW, preterm birth, stillbirth, prenatal death, or abortion (50).

**Cases:** deliveries whose birth outcome was stillbirth, defined as babies born without any signs of life at or after 28 weeks of gestation or at least 1,000 g in weight.

**Controls:** deliveries whose birth outcome was live birth, defined as babies showing evidence of life (such as the beating of the heart or pulsation of the umbilical cord) on delivery at or after 28 weeks of gestation or at least greater than 1,000 g in weight.

### Ethical consideration

The Institutional Review Board of Adama Hospital Medical College's Ethical Committee approved this study. The reference number is SPH/0132/2023. The participants were informed of the purpose of the study, and written informed consent was given by each respondent after the purpose and objectives of the study were explained. Confidentiality and privacy were maintained.

## Results

### Characteristics of the study participants

A total of 403 (81 cases and 322 controls) subjects were included in this study. The average age of the participants was 26 (SD: ± 5.03) years. Of the study subjects, 54 (67%) of cases and 286 (89%) of the controls were urban residents. Regarding the maternal educational status, 28 (34.6%) of cases and 183 (56.8%) of the controls had diplomas. More than half of the study participants were married, with 51 (63%) of the cases and 282 (87.6%) of the controls. Similarly, the majority of the mothers, 68 (83.9%) cases, and 302 (93.8%) of the controls used iron supplements during pregnancy. Concerning the occupational status of the study participants, 35 (43%) of cases and 179 (55.6%) of the controls were housewives (Table 1).

**Table 1:** Characteristics of the women who gave at Bishoftu general hospital 2023.

Variables	Category	Stillbirth		*P* value
		Yes (*n*, %)	No (*n*, %)	
Age (in years)	<20	13 (16.0)	36 (11.2)	0.024
	20–34	58 (71.6)	262 (81.4)	
	>34	10 (12.4)	24 (7.4)	
Residence	Urban	54 (66.7)	286 (88.8)	0.001
	Rural	27 (33.3)	36 (11.2)	
Educational status	Illiterate	18 (22.2)	18 (5.6)	0.001
	Middle school	35 (43.2)	121 (37.6)	
	Diploma & above	28 (34.6)	183 (56.8)	
Marital status	Married	51 (63.0)	282 (87.6)	0.001
	Others^a^	30 (37.0)	40 (12.4)	
Iron and foliate supplement use	Yes	68 (83.9)	302 (93.8)	0.271
	No	13 (16.1)	20 (6.2)	
Occupational status	Housewife	35 (43.2)	179 (55.6)	0.150
	Governmental employees	10 (10.4)	70 (21.7)	
	Others^b^	36 (44.4)	73 (22.7)	
Number of ANC visit	Less than three	43 (53.1)	58 (18.0)	0.030
	Four or more (4^+^)	38 (46.9)	264 (82.0)	
History of prev. stillbirth	Yes	16 (19.7)	11 (3.4)	0.012
	No	65 (80.2)	311 (96.6)	
History of abortion	Yes	14 (17.3)	23 (7.1)	0.021
	No	67 (82.7)	299 (92.9)	
Birth weight	Normal (2,500–4,000 gm)	54 (66.7)	227 (70.5)	0.040
	Non-normal	27 (33.3)	95 (29.5)	
Maternal Rh	Negative	7 (8.6)	10 (3.1)	0.325
	positive	74 (93.8)	312 (96.6)	
History of DM	Yes	5 (6.2)	12 (3.7)	0.452
	No	76 (93.8)	310 (96.3)	

Others.

^a^ Single, divorced, widowed, and separated; others.

^b^ Labor worker, private, merchant.

### Obstetric characteristics of mothers

Regarding the number of ANC visits, 38 (46.9%) cases and 268 (82%) controls had four or more visits, respectively. Sixty-eight (84%) of cases, 302 (93.8%) of the controls had taken iron and foliate supplements. Histories of stillbirth and abortion were recorded; 16 (19.8%) in cases and 11 (3.4%) in controls, 14 (17.3%) cases, and 23 (7.1%) controls had a history of previous stillbirth and abortion,n respectively. Of the total newborns, 54 (66.7%) and 27 (33.3%) of cases had normal and nonnormal birth weights, respectively. Only 5 (6.2%) of cases and 12 (3.7%) of the control had a history of diabetes mellitus (Table 1).

**Figure 1:**
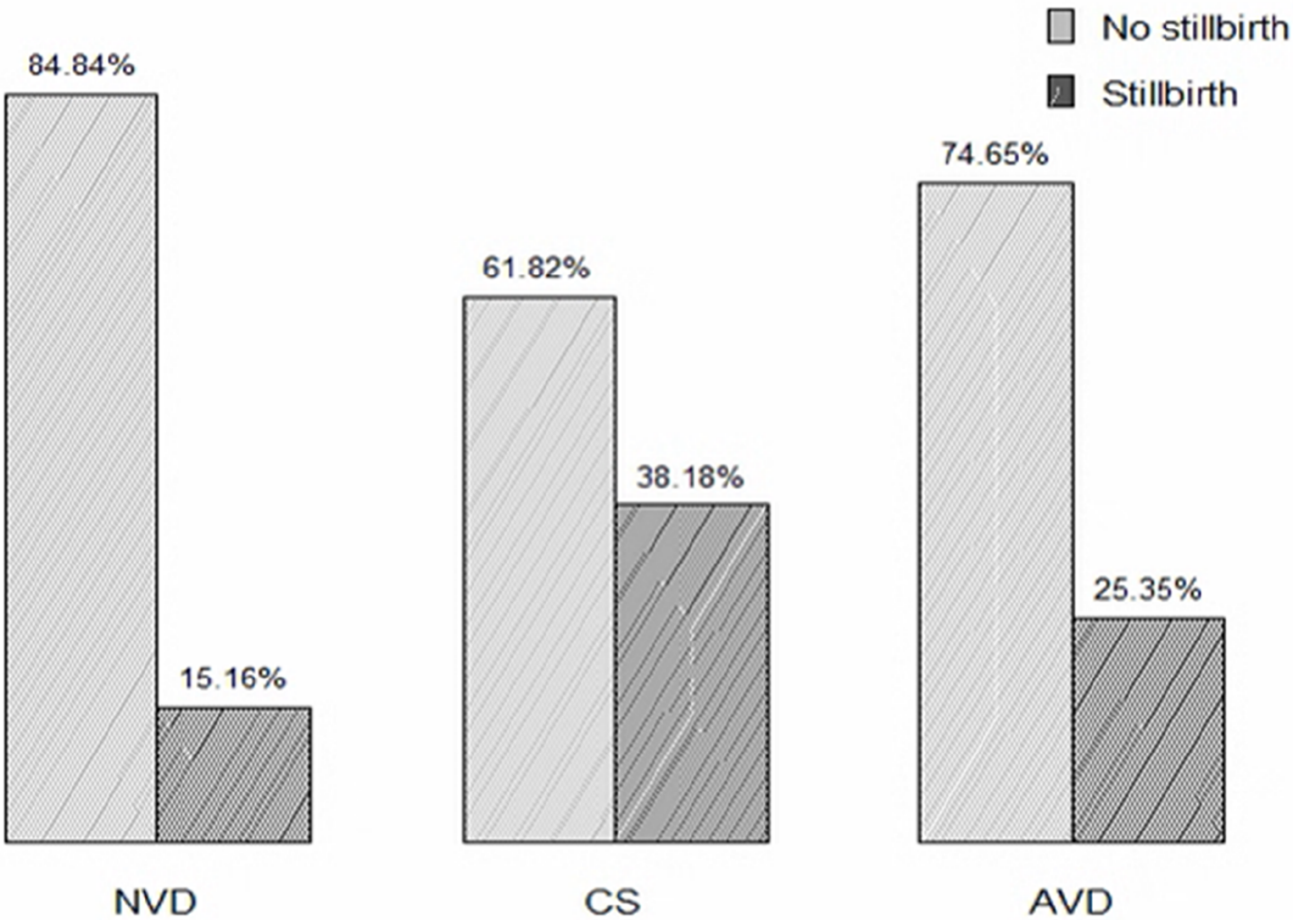
Proportion of stillbirth based on the duration of delivery among mothers who gave birth at Adama hospital medical college.

**Figure 2:**
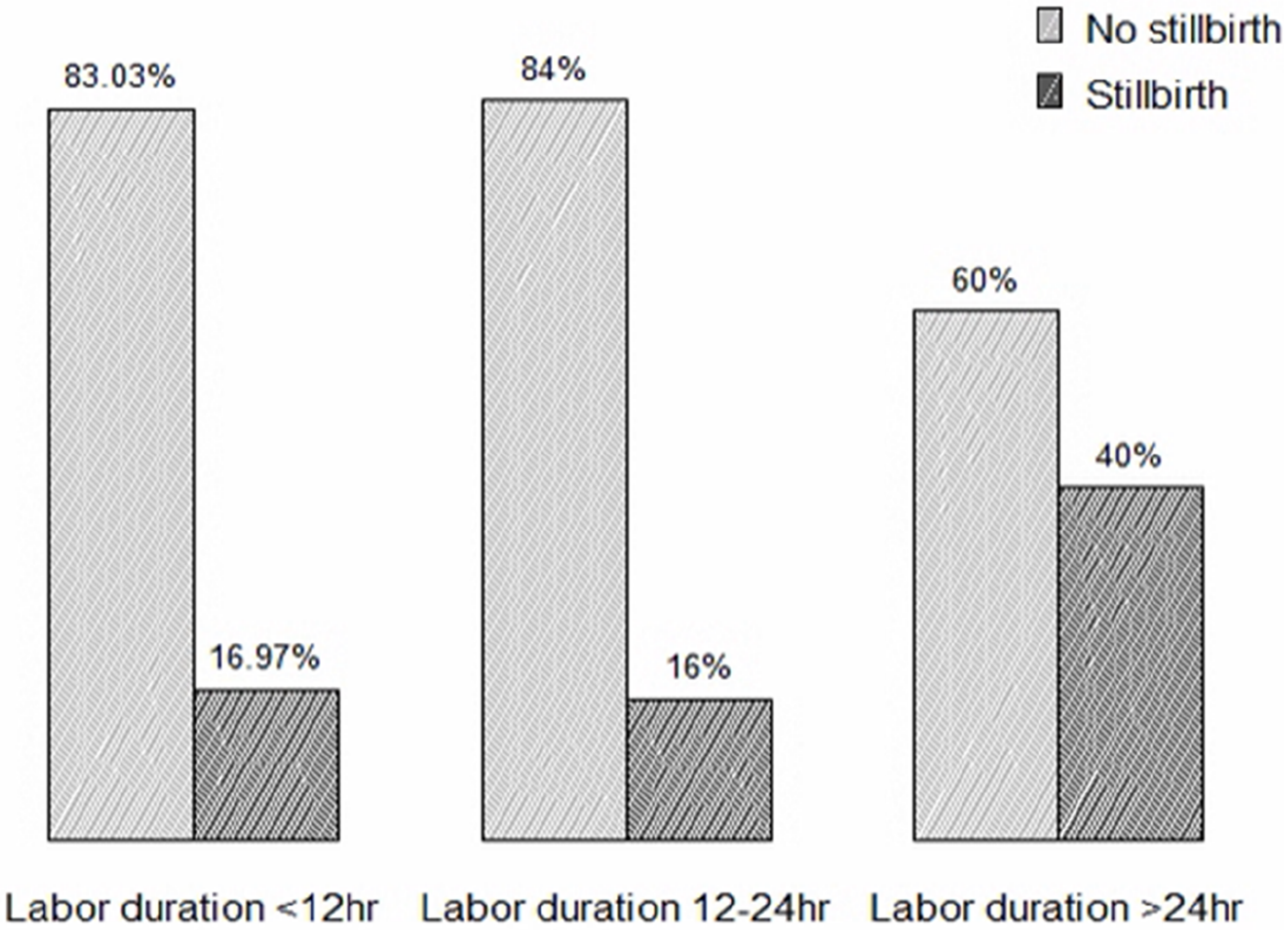
Proportion of stillbirth based on duration labor mothers who gave birth at Adama hospital medical college.

Concerning the mode of delivery, 42 (15.16%), 21 (38.18%), and 24 (25.35%) stillbirths occurred among mothers who delivered via natural vaginal delivery, cesarean section, and assisted vaginal delivery, respectively (Figure 1). Regarding the duration of labor, 37 (16.97%), 20 (16%), and 24 (40%) stillbirths occurred among mothers who labored for less than 12 h, 12–24 h, and more than 24 h, respectively (Figure 2).

### Determinants of stillbirth among women who gave birth

The adjusted odds ratios (AORs) with 95% credible intervals (CrIs) are presented in Table 2. We observed that stillbirth was less likely among women who attained middle school (OR: 0.23; 95% CrI: 0.11, 0.43) and diploma and above school (OR: 0.25; 95% CrI: 0.12, 0.46) than among illiterate women. Women in rural areas were more likely than their urban counterparts to have a stillbirth (OR: 2.55; 95% CrI: 1.11, 4.99). The odds of stillbirth for married women were lower than those of their counterparts (OR: 0.46; 95% CrI: 0.20, 0.93). Women who had more than four ANC visits during the pregnancy were less likely to experience stillbirth than those who had fewer ANC visits (OR: 0.35; 95% CrI: 0.17, 0.63). Women who had a history of previous stillbirths were more likely to have stillbirths (OR: 8.71; 95% CrI: 3.23, 17.69) than their counterparts. The odds of stillbirth of women who had a history of previous abortions were more likely to have stillbirths than their counterparts (OR: 3.29; 95% CrI: 1.13, 7.66). The odds of stillbirth were more likely for women who had labored for more than 24 h than among their counterparts (OR: 3.71, 95% CrI: 25, 7.83). Women who gave birth weighing in the normal range (2,500–4,000 g) were less likely to have a stillbirth than those who had birth in the nonnormal range (OR: 0.32; 95% CrI: 0.15, 0.57).

**Table 2:** Factors associated with stillbirth among mothers who gave birth in this study in bishoftu hospital, oromia regional state, Ethiopia, 2023.

Variables	Category	OR	Std. dev	MCSE	median	95% CrI
Age of mothers	<20	1.0 (ref)				
	20–34	1.792	0.831	0.193	1.581	(0.748, 4.063)
	>34	3.015	2.062	434	2.408	(0.704, 8.424)
Mothers’ education status	Illiterate	1.0 (ref)				
	Middle school	0.232	0.079	0.0122	0.219	(0.113, 0.433)
	Diploma & above	0.249	0.088	0.013	0.233	(0.121, 0.457)
Residence	Urban	1.0 (ref)				
	Rural	2.545	0.992	0.101	2.375	(1.114, 4.997)
Marital status	Others*	1.0 (ref)				
	Married	0.461	0.214	0.036	0.424	(0.204, 0.930)
ANC visits	≤3	1.0 (ref)				
	≥4	0.353	0.118	0.014	0.334	(0.174, 0.633)
History of prev. stillbirth	No	1.0 (ref)				
	Yes	8.709	3.900	0.737	8.068	(3.229, 17.696)
History of prev. abortion	No	1.0 (ref)				
	Yes	3.288	1.663	0.292	2.933	(1.131, 7.658)
Duration of labor	<12 h	1.0 (ref)				
	12–24 h	1.132	0.434	0.054	1.063	(0.522, 2.154)
	>24 h	3.707	1.703	0.275	3.436	(1.247, 7.831)
Mode of delivery	Vaginal delivery	1.0 (ref)				
	Cesarean section	2.194	1.001	0.143	2.039	(0.701, 4.560)
	Assisted vaginal	1.519	0.717	0.152	1.354	(0.630, 3.389)
Birth weight	Non-normal	1.0 (ref)				
	Normal	0.319	0.104	0.016	0.306	(0.150, 0.572)

CrI, credible interval; OR, Odd Ratio; Std.dev, standard deviation; MCSE.

* Divorced/widowed/separated.

## Discussion

This facility-based, unmatched case-control study was conducted among mothers who gave birth at Bishoftu General Hospital to identify determinants of stillbirth. The findings revealed that rural residence, a history of prior stillbirth, a history of abortion, prolonged labor, and birth weight were statistically significant determinants of stillbirth.

Our study found that rural residents were twice as likely to experience stillbirth compared to urban dwellers, aligning with similar findings from Ethiopian (21, 27, 28), India (51), Zimbabwe (52) and Ghana (53). This is because rural residents often have limited access to healthcare, with fewer healthcare facilities, trained professionals, and medical resources, leading to delays in seeking timely prenatal care or medical intervention during complications. Additionally, rural populations tend to face higher rates of poverty (26), lower levels of maternal education (23, 26–28), and inadequate nutrition (28, 54), all of which contribute to a higher risk of stillbirth compared to urban dwellers.

The study found that individuals with a history of stillbirth were significantly more likely to experience a subsequent stillbirth, a finding consistent with studies conducted in India (25, 55), south Africa (55), Nepal (26), and in north western Ethiopia (21, 23). A study suggested that stillbirths in the first two pregnancies may be linked to biological factors, in addition to established risk factors that could affect subsequent pregnancies (56). This may be due to individuals with a prior history may stem from persistent biological or genetic factors (56), such as placental issues or blood disorders, as well as chronic health conditions like hypertension or diabetes (21, 22, 24, 28). Consistent lifestyle factors and psychological stress from a previous stillbirth may further elevate risk (22). Limited healthcare access can also lead to undiagnosed or unmanaged conditions, compounding the risk in future pregnancies.

Women who had previously undergone abortions were threefold more likely to have a stillbirth than their counterparts. There is no underlying mechanism that links a history of past abortions to the causes of stillbirth. However, research performed at the Jimma University referral hospital revealed that bleeding during a prior abortion causes stillbirth in pregnant women (9). The high risk of stillbirth among women with a history of abortion may be due to factors like uterine scarring or cervical insufficiency from prior procedures (21–23), underlying health conditions (21, 24), and recurrent infections (21–23). Persistent lifestyle risks, inadequate prenatal care (9, 26, 29), and the physical and psychological stress following an abortion may also contribute to complications in subsequent pregnancies (22), collectively increasing the risk of stillbirth.

Mothers who labored for more than 24 h had a four-fold higher risk of stillbirth than those who labored for less than 12 h. These findings are consistent with those reported in Ethiopia (8, 57, 58), Bangladesh (59), and Yemen (60). Extended labor can lead to maternal exhaustion and increase the likelihood of complications, such as fetal distress or oxygen deprivation, which elevate the risk of stillbirth. Prolonged labor also raises the chances of infection infections (21–23), uterine rupture, or placental issues (22, 30), which can further compromise fetal well-being. Additionally, lengthy labor may delay necessary medical interventions, especially in settings with limited resources, further increasing the risk of stillbirth outcomes.

Mothers who delivered infants with normal birth weights were less likely to experience stillbirth compared to those who delivered infants with abnormal birth weights. This finding is congruent with previous research conducted in Ethiopia and in Ghana (8, 61). This may be due to abnormal birth weights often indicate underlying issues such as intrauterine growth restriction (IUGR), preterm birth (21, 23, 25, 26), maternal nutrition status (28), and maternal age (5, 25, 28), all of which can increase the risk of stillbirth.

### Limitation of the study

This study has several important limitations. First, it relied on facility-based data, which excluded stillbirths that occurred outside of healthcare settings, such as those in homes or smaller clinics. As a result, the findings may not fully represent stillbirths in the broader population, and the conclusions should be interpreted with caution. Additionally, the study was conducted in a single healthcare facility, which may limit the diversity of the sample and reduce the generalizability of the findings to the entire Ethiopian population. Furthermore, since data was collected over a one-month period, seasonal variations in stillbirth rates were not assessed.

## Conclusion

This study highlights key determinants of stillbirth, including rural residence, prior stillbirth, abortion, prolonged labor, and abnormal birth weights. Addressing these factors through improved healthcare access, maternal education, and targeted interventions could help reduce stillbirth rates, especially in rural and high-risk populations. Further multi-centre research is needed to validate these findings and guide evidence-based policy interventions to effectively reduce stillbirth rates.

## Data Availability

The datasets presented in this study can be found in online repositories. The names of the repository/repositories and accession number(s) can be found in the article/Supplementary Material.

## References

[1] Getiye Y Fantahun M. Factors associated with perinatal mortality among public health deliveries in Addis Ababa, Ethiopia, an unmatched case control study. BMC Pregnancy Childbirth. (2017) 17(1):245. 10.1186/s12884-017-1420-7 28747161 PMC5530490

[2] World Health Organization—Global maternal death rate 2024. Available online at: https://www.who.int/news-room/fact-sheets/detail/maternal-mortality (accessed April 26, 2024).

[3] Khalili N Heidarzadeh M Habibelahi A Tayefi B Ramezani M Rampisheh Z Stillbirth in Iran and associated factors (2014–2016): a population-based study. Med J Islam Repub Iran. (2020) 34:38. 10.34171/mjiri.34.38 32617277 PMC7320973

[4] McClure EM Saleem S Pasha O Goldenberg RL. Stillbirth in developing countries: a review of causes, risk factors and prevention strategies. J Matern Fetal Neonatal Med. (2009) 22(3):183–90. 10.1080/14767050802559129 19089779 PMC3893926

[5] Berhe T Gebreyesus H Teklay H. Prevalence and determinants of stillbirth among women attended deliveries in Aksum General Hospital: a facility based cross-sectional study. BMC Res Notes. (2019) 12:1–6. 10.1186/s13104-018-4038-6 31262356 PMC6604274

[6] Berhan Y Berhan A. Perinatal mortality trends in Ethiopia. Ethiop J Health Sci. (2014) 24:29. 10.4314/ejhs.v24i0.4S 25489181 PMC4249204

[7] Demise A Gebrehiwot Y Worku B Spector JM. Prospective audit of avoidable factors in institutional stillbirths and early neonatal deaths at tikur anbessa hospital in Addis Ababa, Ethiopia. Afr J Reprod Health. (2015) 19(4):78–86. 27337856

[8] Mekonnen Dagne H Takele Melku A Abdurkadir Abdi A. Determinants of stillbirth among deliveries attended in bale zone hospitals, oromia regional state, southeast Ethiopia: a case–control study. Int J Women’s Health. (2021) 13:51–60. 10.2147/IJWH.S276638 33447092 PMC7802824

[9] Tilahun D Assefa TT. Incidence and determinants of stillbirth among women who gave birth in Jimma University specialized hospital, Ethiopia. Pan Afr Med J. (2017) 28:299. 10.11604/pamj.2017.28.299.1269 29721130 PMC5927570

[10] UNICEF W. The Every New Born Action Plan. Ending Preventable newborn deaths and stillbirths by 2030. (2020). Available online at: https://www.unicef.org/media/77166/file/Ending-preventable-newborn-deaths-and-stillbirths-by-2030-universal-health-coverage-in-2020–2025.pdf (accessed September 27, 2016).

[11] WHO. The WHO Application of ICD-10 to Deaths During the Perinatal Period: ICD-PM. World Heal Organ (2016). p. 1–88.

[12] WHO, UNICEF. Reaching the Every Newborn National 2020 Milestones Country Progress, Plans and Moving Forward. Ethiopia: WHO and UNICEF (2017). p. 2015–8. Available online at: http://apps.who.int/iris/bitstream/10665/255719/1/9789241512619-eng.pdf?ua=1

[13] CSA. Ethiopia Mini Demographic and Health Survey. (2019).

[14] UNICEF. UNICEF Briefing Note Series on SDG Global Indicators Related to Children. UNICEF (2015). p. 56–73.

[15] Ethiopia Demographic and Health Survey. (2016). Available online at: https://dhsprogram.com/pubs/pdf/fr328/fr328.pdf (accessed July 10, 2017).

[16] Mini Demographic and Health Survey. (2019). Available online at: https://dhsprogram.com/pubs/pdf/FR363/FR363.pdf (accessed May 20, 2021).

[17] Simegn Alemu Moges Agazhe Yonas Lamore Milkiyas Toru Tefa Birlewu. Prevalence of Still Birth and its Associated Factors in Ethiopia; A Systematic Review and Meta-analysis. Ethiopia: Allied Academies (2021).

[18] Wolde J Haile D Paulos K Alemayehu M Adeko AC Ayza A. Prevalence of stillbirth and associated factors among deliveries attended in health facilities in Southern Ethiopia. PLoS One. (2022) 17(12):e0276220. 10.1371/journal.pone.0276220 36512623 PMC9746959

[19] Lakew D Tesfaye D Mekonnen H. Determinants of stillbirth among women deliveries at Amhara region, Ethiopia. BMC Pregnancy Childbirth. (2017) 17(1):375. 10.1186/s12884-017-1573-4 29132338 PMC5683523

[20] Abdo R Endalemaw T Tesso F. Prevalence and associated factors of adverse birth outcomes among women attended maternity ward at negest elene mohammed memorial general hospital in hosanna town, SNNPR, Ethiopia. J Women’s Health Care. (2016) 5(4):324.

[21] Aregawi G Assefa N Mesfin F Tekulu F Adhena T Mulugeta M Preterm births and associated factors among mothers who gave birth in Axum and Adwa Town public hospitals, Northern Ethiopia, 2018. BMC Res Notes. (2019) 12(1):640. 10.1186/s13104-019-4650-0 31578146 PMC6775657

[22] Aminu M Bar-Zeev S White S Mathai M van den Broek N. Understanding cause of stillbirth: a prospective observational multi-country study from sub-Saharan Africa. BMC Pregnancy Childbirth. (2019) 19:1–10. 10.1186/s12884-019-2626-7 31801488 PMC6894270

[23] Tarekegn WD Worku DG. Determinants of stillbirth in Felege-Hiwot comprehensive specialized referral hospital, North-west, Ethiopia, 2019. BMC Res Notes. (2019) 12(1):579. 10.1186/s13104-019-4085-7 31521188 PMC6744638

[24] Barrett PM McCarthy FP Evans M Kublickas M Perry IJ Stenvinkel P Stillbirth is associated with increased risk of long-term maternal renal disease: a nationwide cohort study. Am J Obstet Gynecol. (2020) 223(3):427.e1–e14. 10.1016/j.ajog.2020.02.031 32112729 PMC7479504

[25] Newtonraj A Kaur M Gupta M Kumar R. Level, causes, and risk factors of stillbirth: a population-based case control study from Chandigarh, India. BMC Pregnancy Childbirth. (2017) 17:1–9. 10.1186/s12884-017-1557-4 29132325 PMC5684767

[26] Kc A Wrammert J Ewald U Clark RB Gautam J Baral G. Incidence of intrapartum stillbirth and associated risk factors in tertiary care setting of Nepal: a case-control study. Reprod Health. (2016) 13:1–11. 10.1186/s12978-016-0226-9 27581467 PMC5007702

[27] Gebremeskel F Gultie T Kejela G Hailu D Workneh Y. Determinants of adverse birth outcome among mothers who gave birth at hospitals in Gamo Gofa Zone, Southern Ethiopia: a facility based case control study. Qual Prim Care. (2017) 25(5):259–66. 10.34763/jmotherandchild.20212501

[28] Berhie KA Gebresilassie HG. Logistic regression analysis on the determinants of stillbirth in Ethiopia. Matern Health Neonatol Perinatol. (2016) 2:1–10. 10.1186/s40748-016-0038-5 27660718 PMC5025573

[29] Liu LC Wang YC Yu MH Su HY. Major risk factors for stillbirth in different trimesters of pregnancy—a systematic review. Taiwan J Obstet Gynecol. (2014) 53(2):141–5. 10.1016/j.tjog.2014.04.003 25017256

[30] Shattnawi KK Khader YS Alyahya MS Al-Sheyab N Batieha A. Rate, determinants, and causes of stillbirth in Jordan: findings from the Jordan Stillbirth and Neonatal Deaths Surveillance (JSANDS) system. BMC Pregnancy Childbirth. (2020) 20(1):571. 10.1186/s12884-020-03267-2 32993562 PMC7526247

[31] Muglu J Rather H Arroyo-Manzano D Bhattacharya S Balchin I Khalil A Risks of stillbirth and neonatal death with advancing gestation at term: a systematic review and meta-analysis of cohort studies of 15 million pregnancies. PLoS Med. (2019) 16(7):e1002838. 10.1371/journal.pmed.1002838 31265456 PMC6605635

[32] Federal Ministry of Health, Ethiopia. Ethiopian National Newborn and Child Survival Strategy. Ethiopia: Federal Ministry of Health (2015).

[33] Federal Ministry of Health, Ethiopia. Maternal and Child Health Quality Improvement Plan. Ethiopia: Federal Ministry of Health (2018).

[34] Tura G Fantahun M Worku A. The effect of health facility delivery on neonatal mortality: systematic review and meta-analysis. BMC Pregnancy Childbirth. (2013) 13(1):18. 10.1186/1471-2393-13-18 23339515 PMC3584809

[35] Gebrehiwot T Goicolea I Edin K San Sebastian M. Making pragmatic choices: women’s experiences of delivery care in northern Ethiopia. BMC Pregnancy Childbirth. (2012) 12(1):113. 10.1186/1471-2393-12-113 23078068 PMC3542090

[36] Gebremeskel GG Berhe KK Belay DS Kidanu BH Negash AI Gebreslasse KT Magnitude of metabolic syndrome and its associated factors among patients with type 2 diabetes mellitus in ayder comprehensive specialized hospital, Tigray, Ethiopia: a cross sectional study. BMC Res Notes. (2019) 12(1):603. 10.1186/s13104-019-4609-1 31533851 PMC6751785

[37] Gemeda D Abebe E Duguma A. Metabolic syndrome and its associated factors among type 2 diabetic patients in Southwest Ethiopia, 2021/2022. J Diabetes Res. (2022) 2022:8162342. 10.1155/2022/8162342 36248224 PMC9553723

[38] Shita A Teshome H Ayalew M Yesuf W Getachew D. Metabolic syndrome and its associated factors among type 2 diabetic patients in Mizan-Tepi university teaching hospital, Southwest Ethiopia Region. Front Clin Diabetes Healthc. (2023) 4:1234674. 10.3389/fcdhc.2023.1234674 37790676 PMC10542573

[39] Tadewos AAH Assegu D. Pattern of metabolic syndrome in relation to gender among type-II DM patients in hawassa university comprehensive specialized hospital, Hawassa, Southern Ethiopia. Health Sci J. (2017) 11:3.

[40] Zerga AA Bezabih AM. Metabolic syndrome and lifestyle factors among type 2 diabetes mellitus patients in dessie referral hospital, Amhara region, Ethiopia. PLoS One. (2020) 15(11):e0241432. 10.1371/journal.pone.0241432 33137150 PMC7605694

[41] Almasi-Hashiani A Sepidarkish M Safiri S Khedmati Morasae E Shadi Y Omani-Samani R. Understanding determinants of unequal distribution of stillbirth in Tehran, Iran: a concentration index decomposition approach. BMJ Open. (2017) 7(5):e013644. 10.1136/bmjopen-2016-013644 28515186 PMC5777464

[42] Endeshaw G Berhan Y. Perinatal outcome in women with hypertensive disorders of pregnancy: a retrospective cohort study. Int Sch Res Notices. (2015) 2015:208043. 10.1155/2015/208043 27347505 PMC4897150

[43] Amegah AK Näyhä S Jaakkola JJ. Do biomass fuel use and consumption of unsafe water mediate educational inequalities in stillbirth risk? An analysis of the 2007 Ghana maternal health survey. BMJ Open. (2017) 7(2):e012348. 10.1136/bmjopen-2016-012348 28174221 PMC5306511

[44] Zeitlin J Mortensen L Prunet C Macfarlane A Hindori-Mohangoo AD Gissler M Socioeconomic inequalities in stillbirth rates in Europe: measuring the gap using routine data from the euro-peristat project. BMC Pregnancy Childbirth. (2016) 16(1):15. 10.1186/s12884-016-0804-4 26809989 PMC4727282

[45] Siyoum M Melese T. Factors associated with low birth weight among babies born at Hawassa university comprehensive specialized hospital, Hawassa, Ethiopia. Ital J Pediatr. (2019) 45(1):48. 10.1186/s13052-019-0637-7 30975170 PMC6460807

[46] Spiegelhalter DJ Bayesian Methods in Health Technology Assessment: A Review. Cambridge, UK: NIHR Journals Library (2000). 11134920

[47] Gelman A Carlin JB Stern HS Dunson DB Vehtari A Ruben DB. Bayesian Data Analysis. New York: Chapman and Hall/CRC (1995).

[48] Presern C Bustreo F Lawn J. Born too Soon: The Global Action Report on Preterm Birth. Geneva: World Health Organization (2012). p. 1. Available online at: https://press.un.org/en/2012/120502_who.doc.htm (accessed March 20, 2023).

[49] Aminu M UR Mdegela M Utz B Adaji S Van Den Broek N. Causes of and factors associated with stillbirth in low-and middle-income countries: a systematic literature review. BJOG. (2014) 121:141–53. 10.1111/1471-0528.12995 25236649

[50] Kallela J The diagnosis of pre-eclampsia using two revised classifications in the Finnish Pre-eclampsia consortium (FINNPEC) cohort. BMC Pregnancy Childbirth. (2016) 16:221. 10.1186/s12884-016-1010-0 27520381 PMC4983019

[51] Patel KK Saroj RK Kumar M. Prevalence and determinants of adverse pregnancy outcomes among women in India: a secondary data analysis. Indian J Community Med. (2021) 46(3):434–7. 10.4103/ijcm.IJCM_627_20 34759482 PMC8575229

[52] Serie L. Stillbirths an executive summary for the lancet series. Health Syst Res Unit. (2011) 10.

[53] Löfwander M. Stillbirths and Associations with Maternal Education. A Registry Study from a Regional Hospital in North Eastern Tanzania. Norway: University of Tromsø (2012).

[54] Zakar MZ Zakar R Mustafa M Jalil A Fischer F. Underreporting of stillbirths in Pakistan: perspectives of the parents, community and healthcare providers. BMC Pregnancy Childbirth. (2018) 18:1–9. 10.1186/s12884-018-1924-9 30012104 PMC6048905

[55] Mony PK Varghese B Thomas T. Estimation of perinatal mortality rate for institutional births in rajasthan state, India, using capture–recapture technique. BMJ Open. (2015) 5(3):e005966. 25783418 10.1136/bmjopen-2014-005966 PMC4369003

[56] Hossain N Khan N Khan NH. Obstetric causes of stillbirth at low socioeconomic settings. J Pak Med Assoc. (2009) 59(11):744–7. 20361671

[57] Gizaw W Feyisa M Hailu D Nigussie T. Determinants of stillbirth in hospitals of North Shoa Zone, Oromia region, central Ethiopia: a case control study. Heliyon. (2021) 7:e07070. 10.1016/j.heliyon.2021.e07070 34041408 PMC8141871

[58] Welegebriel TK Dadi TL Mihrete KM. Determinants of stillbirth in Bonga general and Mizan Tepi university teaching hospitals southwestern Ethiopia, 2016: a case–control study. BMC Res Notes. (2017) 10. 10.1186/s13104-017-3058-y 29301566 PMC6389129

[59] Nahar S Rahman A Nasreen HE. Factors influencing stillbirth in Bangladesh: a case-control study. Paediatr Perinat Epidemiol. (2013) 27(2):158–64. 10.1111/ppe.12026 23374060

[60] Obadi MA Taher R Qayad M Khader YS. Risk factors of stillbirth in Yemen. J Neonatal Perinatal Med. (2018) 11(2):131–6. 10.3233/NPM-181746 29843265

[61] Omo-Aghoja LO Onohwakpor EA Adeyinka AT Omene JA. Incidence and determinants of stillbirth amongst parturients in two hospitals in southern Nigeria. J Basic Clin Reprod Sci. (2014) 3:15. 10.4103/2278-960X.129273

